# Improvement of Hydrophilicity for Polyamide Composite Membrane by Incorporation of Graphene Oxide-Titanium Dioxide Nanoparticles

**DOI:** 10.1155/2020/6641225

**Published:** 2020-12-30

**Authors:** Thu Hong Anh Ngo, Chau Thi Minh Nguyen, Khai Dinh Do, Quan Xuan Duong, Nghia Hieu Tran, Hoan Thi Vuong Nguyen, Dung Thi Tran

**Affiliations:** ^1^Faculty of Chemistry, University of Science, Vietnam National University, Hanoi, Vietnam; ^2^Faculty of Chemistry, Quy Nhon University, Quy Nhon, Vietnam

## Abstract

In this work, the polyamide (PA) membrane surface has been modified by coating of nanomaterials including graphene oxide (GO) and titanium dioxide (TiO_2_) to enhance membrane separation and antifouling properties. The influence of surface modification conditions on membrane characteristics has been investigated and compared with a base membrane. Membrane surface properties were determined through scanning electron microscope (SEM) images and Fourier transform infrared-attenuated total reflectance (FTIR-ATR) spectroscopy. Membrane separation performance was determined through the possibility for the removal of methylene blue (MB) in water. Membrane antifouling property was evaluated by the maintained flux ratios (%) after 120 minutes of filtration. The experimental results showed that the appearance of hydrophilic groups after coating of GO and TiO_2_ nanocomposite materials with or without UV irradiation onto membrane surface made an improvement in the separation property of the coated membranes. The membrane flux increased from 28% to 61%; meanwhile, the antifouling property of the coated membranes was improved clearly, especially for UV-irradiated PA/GO-TiO_2_ membrane.

## 1. Introduction

Along with the rapid economic development, water pollution is becoming a serious problem. Recently, the demand for use of membrane technology including ultrafiltration and/or nanofiltration and reverse osmosis is emerging in wastewater treatment and ultrapure water production [1].

One of the most commonly used commercial membranes in those applications is the polyamide composite (PA) membrane [2]. This composite membrane is made of a thin-film polyamide top layer and a polysulfone sublayer [2, 3]. Moreover, this kind of membrane has high permeability, flux and ion rejection, high resistance to pressure and temperature, pH compaction, and high stability to biological attack [3]. However, the application of PA membrane is limited by the hydrophobicity of the polyamide skin layer, leading to membrane fouling phenomenon, reduction in separation performance, and increase of operation cost [1–3].

To the best of our knowledge, the fouling phenomenon could be reduced by incorporating hydrophilic agents in/onto the membrane surfaces [1–4]. These hydrophilicity materials can reduce the adsorption or adhesion of foulants at the surface of the membrane [4].

Recently, the increase in hydrophilicity or antifouling property of membrane in general and PA membrane in particular has been carried out through chemical grafting, physical blending, or physical coating [4–14]. Among these methods, the coating of hydrophilic agents such as mesoporous silica, titanium dioxide (TiO_2_) [5–8], zinc oxide (ZnO) [9], or graphene oxide (GO) [10–14] onto membrane surface is one of the useful methods to enhance membrane antifouling property. In which, graphene oxide and titanium dioxide are the two most common hydrophilic agents that could be used to modify membrane surfaces [15–19]. GO material has an easy forming nanosheet layer. This layer makes the pore size quite narrow. So, when a rigid material (such as TiO_2_ and ZnO) is inserted into this layer, the GO film will be difficult to form and GO particles will most likely be coated onto the membrane by a physical bond [15].

Xu et al. [15] first synthesized a GO-TiO_2_ composite membrane via vacuum filtration to remove dyes (methyl orange and rhodamine B) from water to evaluate the adsorption and purification capacity of organic wastewater. Wang et al. [18] prepared the TiO_2_@GO-incorporated membranes by interfacial polymerization and embedding TiO_2_@GO nanocomposite in the polyamide layer. The experimental results showed that the 0.2 wt % TiO_2_@GO-modified membrane had an enhancing permeability due to the presence of the TiO_2_@GO nanocomposite material. In the previous paper [19], we used graphene oxide-titanium dioxide (GO-TiO_2_) mixture by a physical blending method for modification of the polysulfone membrane. Experimental results showed that, due to the appearance of oxygen-containing groups of GO-TiO_2_ mixture, these blended membranes become more hydrophilic and have high separation and antifouling properties.

However, the modification of commercial membrane surfaces are being used to reduce solvent and fabrication costs, and thus, the method of membrane surface modification by graft polymerization or physical coating is increasingly used. In our previous paper [20], we modified the PA membrane by coating TiO_2_ nanoparticles onto the membrane surface. The experimental results showed that the separation performance of coated membranes was improved, especially under UV irradiation, with the flux enhancement and the higher maintained flux ratios. However, when the filtration time was extended, the fouling phenomenon was quite rapid. In addition, titanium dioxide nanoparticles were aggregated easily onto the PA membrane surface.

So, in order to decrease the TiO_2_ aggregation onto the membrane and to improve the antifouling performance, in this study, graphene oxide-titanium dioxide nanomaterial was coated onto the polyamide thin-film composite membrane. Graphene oxide is a hydrophilic material. By incorporation of both graphene oxide and titanium dioxide onto the PA membrane surface, the antifouling property of the PA membrane surface will be improved.

## 2. Experimental Methods

### 2.1. Materials

A commercial PA membrane (Filmtec TW30) was used as the substrate. The membrane samples were cut and carefully soaked in a 25 v/v % aqueous solution of isopropanol (purity 99.9%, Sigma-Aldrich) for 60 min, rinsed with deionized water, and kept wet until using for coating. Graphene oxide (GO) was prepared from graphite powder (Merck) with the Hummers method [21] and the commercial TiO_2_ nanoparticles (Merk) were used for the preparation of the coating solution. Methylene blue (MB) (China) has been used to prepare feed solutions for membrane filtration experiments.

### 2.2. Modification of PA Membranes

The solutions of GO or TiO_2_ or GO-TiO_2_ materials in suspension were prepared by the ultrasonic method. To modify the PA membrane surface, the base membrane was placed in a membrane cell and a suspension solution of GO or TiO_2_ or GO-TiO_2_ was compressed through the membrane at a specified pressure. Then, the coated membrane was rinsed carefully with deionized water and/or exposed to UV light (254 nm, 32 W) at different times.

### 2.3. Characteristics of Materials

The surface morphology, size, and elemental mapping of the GO-TiO_2_ samples were determined through electron microscopy (SEM-EDX, Nova NanoSEM 450) and transmission electron microscopy (TEM, JEOL 2100F). Fourier transform infrared spectroscopy method (FTIR-ATR) was used to confirm the presence of hydrophilic agents for GO-TiO2 samples which were recorded on Perkin Elmer spectrophotometer.

### 2.4. Characteristics of Membrane Surface

The scanning electron microscopy (SEM) was used to determine the membrane surface morphology, using a field-emission scanning electron microscope (FE-SEM, Hitachi S-4800). The Fourier transform infrared spectroscopy-attenuated total reflectance (FTIR-ATR, Spectro100 Perkin Elmer) was used to evaluate the surface chemical functionality of the membranes. All membrane samples were dried at 25°C under vacuum before characterization.

### 2.5. Assessment of Membrane Separation Properties

The membrane separation experiments were performed in a dead-end membrane system, supplied by Osmonics (USA), through a membrane area of 13.2 cm^2^. Filtration experiments were carried out at room temperature. The membranes were filtrated by deionized water at 15 bar for 15 min before carrying out the filtration measurements.

Membrane separation performance was determined through permeability *J*_w_ (L.m^−2^.h^−1^.bar^−1^) = (*V*_w_/(A.t.P)), where *V*_w_ is the volume of pure water gained through the membrane area of A under the pressure of P. Ratio of permeability (*J*_w_/*J*_wo_), where *J*_w_ and *J*_wo_ are the permeability of the coated and uncoated membranes, respectively, was used to examine the enhanced permeability of the membrane. The flux (*J*, L.m^−2^.h^−1^) is evaluated by *J* = [*V*/(A.t.), where *V* is the volume of the filtrate through the membrane. The flux ratio (*J*/*J*_o_) has been used to determine the improvement of the flux of coated membranes, in which *J* and *J*_o_ are the fluxes of the coated and uncoated membranes, respectively. The retention (*R*) for the removal of methylene blue is evaluated by *R* = ((*C*_o_−*C*)/*C*_o_)^*∗*^100 (%), where *C*_0_ and *C* are the concentrations of methylene blue in feed and filtrate, respectively.

### 2.6. Evaluation of Membrane Antifouling Property

The membrane antifouling property was determined through the maintained flux ratios (FM, %) during filtration. The maintained flux ratio was evaluated by FM = (*J*_*t*_/*J*_to_)^*∗*^100 (%), where *J*_*t*_ and *J*_to_ are the fluxes of membranes at *t* time and initial time (L.m^−2^.h^−1^).

## 3. Results and Discussion

### 3.1. Characteristics of GO-TiO_2_ Nanoparticles

In the previous work [19], the successful preparation of GO-TiO_2_ materials had been confirmed through a scanning electron microscope (SEM), energy dispersive X-ray spectrometry mapping (EDX mapping), transmission electron microscope (TEM) images, and Fourier transform infrared spectral analysis (FTIR).

**Figure 1:**
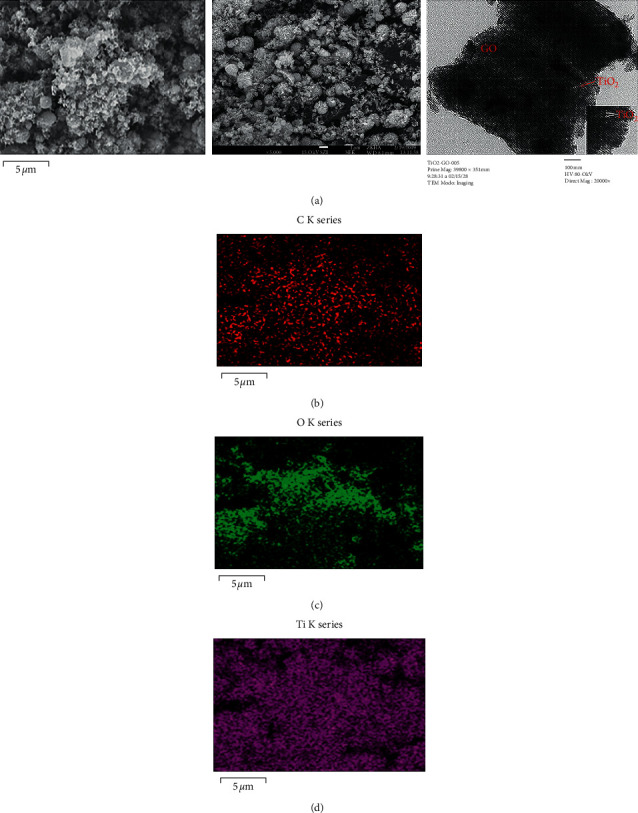
SEM (a1, a2), TEM (a3), and EDX mapping images of C (b), O (c), and Ti (d) elements of GO-TiO_2_ nanocomposite materials.

SEM images (Figures 1(a1) and 1(a2)) showed that the TiO_2_ nanoparticles appeared in the GO matrix. TEM image of GO-TiO_2_ materials showed that the TiO_2_ particles dispersed onto the GO sheets had an average particle size of 10 nm (Figure 1(a3)). So, GO-TiO_2_ material was synthesized successfully. Moreover, using EDX mapping images, the components in the GO-TiO_2_ mixture are determined in Table 1. Figures 1(b))–1(d)) show that there is a homogeneous distribution of the C, O, and Ti elements in the prepared materials.

**Figure 2:**
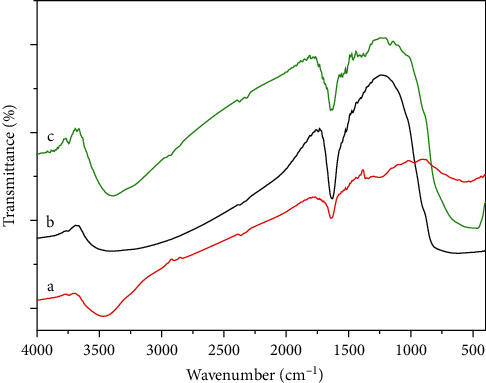
FTIR spectra of GO (curve a), TiO_2_ (curve b), and GO-TiO_2_ (curve c) materials.

The FTIR-ATR spectra of GO, TiO_2_, and GO-TiO_2_ materials are shown in Figure 2. The peaks of GO (curve a in Figure 2) were observed by the appearance of hydrophilic groups including C−O stretching (1050 cm^−1^), C=O stretching (1720 cm^−1^), and O−H stretching (3350 cm^−1^). Moreover, the spectrum of the TiO_2_ (curve b in Figure 2) and GO-TiO_2_ mixture (curve c in Figure 2) shows peaks at approximately 580–1000 cm^−1^ which were assigned to the Ti–O-Ti stretching [19], showing the successful synthesis of GO-TiO2 material.

### 3.2. Membrane Characteristics

#### 3.2.1. SEM Images

**Figure 3:**
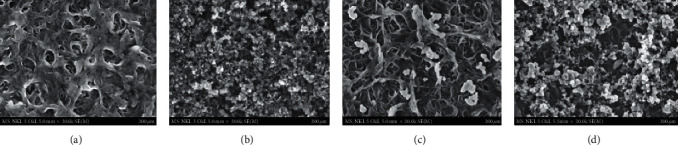
SEM images of (a) PA, (b) PA/GO, (c) PA/TiO_2_, and (d) PA/GO-TiO_2_ membrane surfaces.

SEM images of uncoated polyamide (PA) and coated PA/GO, PA/TiO_2_, and PA/GO-TiO_2_ membranes are displayed in Figure 3. The concentrations of GO and TiO_2_ nanoparticles in the coated solutions are 4 ppm and 35 ppm, respectively.

Because GO material has an easy forming nanosheet layer in the GO and GO-TiO2 coated membranes [15], TiO2 particles in the matrix GO-TiO2 material are more evenly distributed in the GO sheets. Because of the presence of this layer, TiO_2_ particles in the matrix GO-TiO_2_ material are more evenly distributed in the GO sheets. Meanwhile, for the TiO_2_-coated membrane, TiO_2_ particles are easily aggregated, which affects the surface properties of the membrane.

The formation of materials containing both GO and TiO_2_ onto PA membrane surfaces can lead to changes in membrane chemical functionality and membrane separation performance.

#### 3.2.2. FTIR-ATR Spectrum

**Figure 4:**
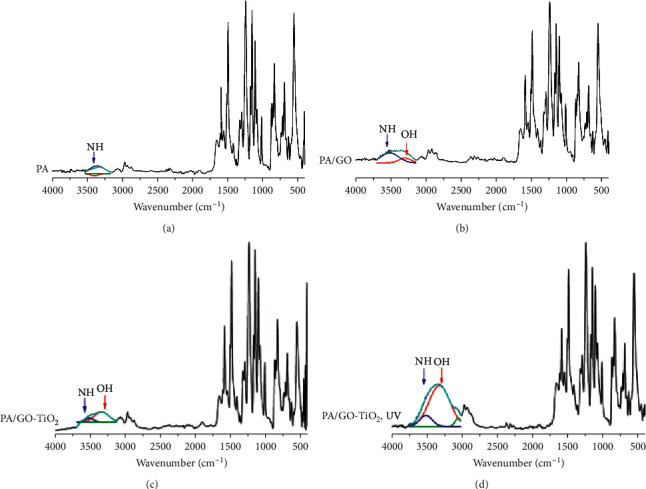
FTIR-ATR spectra of (a) PA, (b) PA/GO, (c) PA/GO-TiO_2_ without UV light, and (d) PA/GO-TiO_2_ with UV light membranes.

In Figure 4, the FTIR-ATR spectroscopy was used to characterize the membrane chemical functionality. The spectrum of the uncoated PA membrane revealed absorptions of N-H (3350 cm^−1^), C-H (2950 cm^−1^), C=O (1650 cm^−1^), C=C (1500 cm^−1^), and C-N (1200 cm^−1^). Further analysis of the spectrum of the PA-, PA/GO-, and PA/GO-TiO_2_-coated membrane surfaces without and with UV light confirmed the presence of one absorption of N-H stretching at 3330 cm^−1^ for the uncoated PA membrane and the appearance of two absorptions of O-H stretching at 3219 cm^−1^ and N-H stretching at 3317 cm^−1^ on the PA/GO-TiO_2_-coated membrane. For PA/GO, PA/GO-TiO_2_, and PA/GO-TiO_2_/UV membranes, the absorbance intensity at the 3219 cm^−1^ (O-H group) increases, showing an increasing hydrophilic level, especially for the PA/GO-TiO_2_-coated membrane under UV irradiation. So, FTIR-ATR spectra demonstrate the successful incorporation of hydrophilic GO and TiO_2_ materials onto the PA membrane surface.

### 3.3. Membrane Separation Property

Indeed, the appearance of the hydrophilic groups of GO and TiO_2_ materials after modification makes the coated membranes become more hydrophilic, and the experimental results related to membrane filtration performance are shown as follows.

#### 3.3.1. Effect of the GO Concentration on the Coated Membrane Filtration Performance

The permeability and flux can be used to characterize the changes in the hydrophilicity of the membrane surface. These parameters will be increased if the membrane surfaces become more hydrophilic [14].

**Figure 5:**
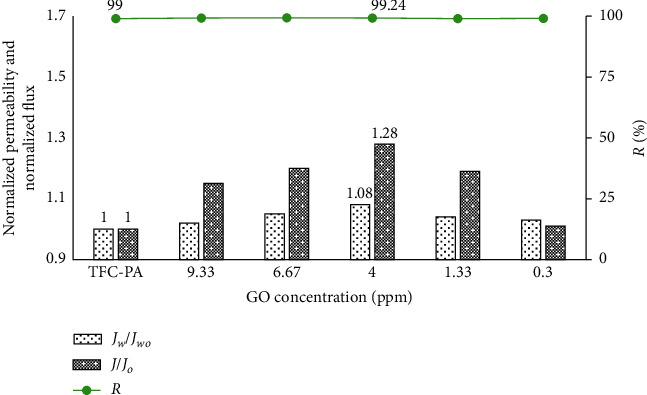
Normalized permeability and normalized flux of the uncoated and GO-coated membranes.

**Figure 6:**
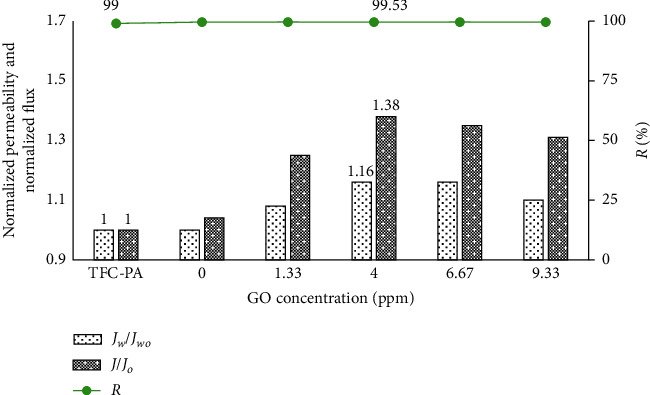
Normalized permeability and normalized flux of the uncoated and GO-TiO_2_-coated membranes without UV irradiation.

**Figure 7:**
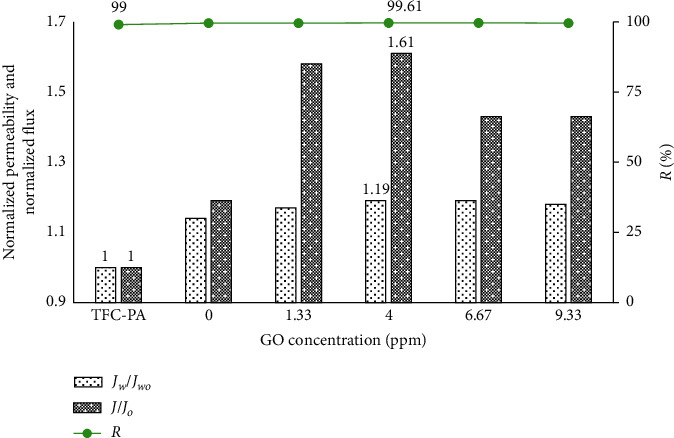
Normalized permeability and normalized flux of the uncoated and GO-TiO_2_-coated membranes with UV irradiation.

In this experiment, the membrane was placed in a dead-end membrane filtration system, and the GO and GO-TiO_2_ mixture in suspension were prepared with a concentration of TiO_2_ of 35 ppm (the TiO_2_ optimal concentration in our previous work) and different concentrations of GO (from 0.30 ppm to 9.33 ppm). After ultrasonic vibrations, the coated membranes were compressed for 3 minutes at a pressure of 12 bar. Then, the membranes were carefully washed with deionized water. The experimental results of membrane separation properties are shown in Figures 5–7.

The experimental results showed that the methylene blue retention of all coated membranes was equivalent to the base membrane. The permeability and flux of all coated membranes could increase with the increase in GO concentration and are higher than those of the uncoated membrane due to the hydrophilic groups onto the membrane surface after coating, especially under UV irradiation. At GO concentration of 4 ppm, the permeability and flux reached the maximum value. The higher concentration of GO-TiO_2_ in the suspension makes an increase in the density of self-assembled GO-TiO_2_ on the membrane surface, so membrane surface resistance could be increased, thus reducing the membrane flux.

From the experimental results, we also found that the water permeability and filtration capacity of the GO-TiO_2_-coated membranes were significantly improved compared to the GO-coated membranes, especially under UV irradiation. Indeed, under UV irradiation, the strengthening in intensity of the hydrophilic OH functional group onto PA/GO-TiO2 membranes was also shown in the FTIR-ATR images. Therefore, the membrane surface becomes more hydrophilic, and thus, the antifouling property and the separation performance of coated membranes will be improved.

#### 3.3.2. Antifouling Property

**Figure 8:**
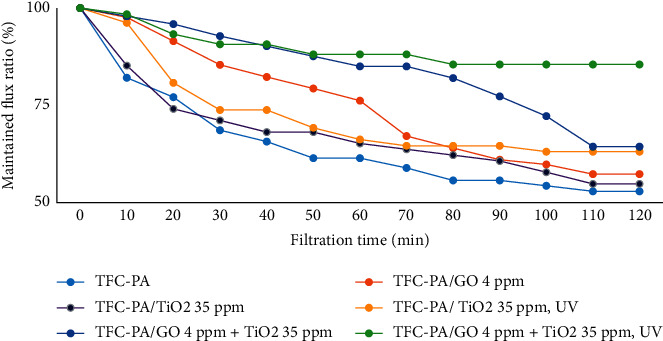
Maintained flux ratios of the uncoated and coated membranes.

Figures 8 shows the differences in the maintained flux ratios between uncoated and coated membranes. The results indicate that the maintained flux values of all coated membranes are increased compared to the uncoated membrane. After 120 min of filtration of the methylene blue solution, the maintained flux ratio of the uncoated membrane was about 52.9%; meanwhile, it was higher than 55% for the coated membranes; especially for the coated membranes under UV irradiation, these membranes have a much higher filtration, up to more than 60 and 80%. Thus, it can be seen that the coating of GO, TiO_2,_ or GO-TiO_2_ particles onto the PA membrane surface significantly increases the membrane antifouling.

## 4. Conclusions

In this work, the PA membrane surface was successfully modified by coating of GO and TiO_2_ nanoparticles. The hydrophilicity of the coated membranes was improved in comparison with that of the uncoated ones.

The results of membrane separation evaluation showed that the methylene blue retention of the coated membranes was maintained well; meanwhile, the flux and antifouling property of all coated membranes had a simultaneous increase, especially for the UV-irradiated PA/GO-TiO_2_-coated membrane.

## Figures and Tables

**Table 1:** The components in the prepared GO-TiO_2_ mixture.

Elements	C	O	Ti
Percentage	14.22	54.79	30.99

## Data Availability

All the data and supporting materials are included within the paper.
